# NLRP3 Inflammasome in the Pathogenesis of Miscarriages

**DOI:** 10.3390/ijms251910513

**Published:** 2024-09-29

**Authors:** Wioleta Justyna Omeljaniuk, Marzena Garley, Anna Pryczynicz, Joanna Motyka, Angelika Edyta Charkiewicz, Elżbieta Milewska, Piotr Laudański, Wojciech Miltyk

**Affiliations:** 1 Department of Analysis and Bioanalysis of Medicines, Medical University of Bialystok, Mickiewicza 2D, 15-222 Bialystok, Polandwojciech.miltyk@umb.edu.pl (W.M.); 2 Department of Immunology, Medical University of Bialystok, Waszyngtona 15A, 15-269 Bialystok, Poland; 3 Department of Pathomorfology, Medical University of Bialystok, Waszyngtona 13, 15-269 Bialystok, Poland; anna.pryczynicz@umb.edu.pl; 4 Independent Researcher, 15-089 Bialystok, Poland; 5 Department of Clinical Molecular Biology, Medical University of Bialystok, Waszyngtona 13, 15-269 Bialystok, Poland; 6 Department of Obstetrics, Gynecology and Gynecological Oncology, Medical University of Warsaw, Kondratowicza 8, 03-242 Warsaw, Poland; 7 Women’s Health Research Institute, Calisia University, Nowy Świat 4, 62-800 Kalisz, Poland; 8 OVIklinika Infertility Center, Połczyńska 31, 01-377 Warsaw, Poland

**Keywords:** NLRP3, IL-1β, IL-18, CYCS, Caspase-1, Fas, FasL, Bcl-2, Ca, K, Mg, Na, miscarriage, apoptosis, NETosis, inflammasome

## Abstract

Despite significant advances in prenatal medicine, spontaneous miscarriage remains one of the most common and serious pregnancy complications, affecting an increasing number of women. Since many aspects of the pathogenesis of spontaneous miscarriage remain unexplained, the aim of this study has been to assess the involvement of the NLRP3 inflammasome as a potential causative factor. The concentrations of NLRP3, IL-1β, IL-18, and cytochrome C in the serum of patients after miscarriage were measured by means of the immunoenzymatic method. In the placental tissue, the expression of NLRP3, IL-1β, IL-18, and Caspase-1 as well as that of the classical apoptosis biomarkers Fas, FasL, Bcl-2, and Ca was evaluated by means of immunohistochemistry techniques. Additionally, in whole blood, the concentrations of elements crucial for pregnancy progression, such as Ca, K, Mg, and Na, were examined by means of the ICP-OES method. Significantly higher concentrations of NLRP3 and IL-18 were demonstrated in the serum of patients with miscarriage as compared to the control group. In the placental tissue samples, a higher expression of IL-1β, IL-18, and Caspase-1 proteins was noted in women who had experienced miscarriage as compared to the control group. At the same time, a significantly lower expression of FasL and Bcl-2 proteins as well as Ca deposits was observed in women after miscarriage as compared to those with a normal pregnancy outcome. Significantly lower concentrations of Ca and K were recorded in the blood of patients with spontaneous miscarriage as compared to pregnant women. The analysis of the results x indicated a greater involvement of the inflammasome in women with spontaneous miscarriage associated with oxidative–antioxidative imbalance than in the case of miscarriage related to NET formation. Our research has provided evidence for the involvement of the inflammasome in the process of spontaneous miscarriage and identifies a new direction for diagnostics that includes NLRP3 as a preventive element in prenatal care, particularly in light of the steadily declining number of pregnancies and the increasing number of reproductive failures.

## 1. Introduction

Spontaneous miscarriage is the most common pregnancy complication, being characterised by the spontaneous expulsion of the entire embryo or its fragments from the uterine cavity during a period when the foetus is not yet viable outside the mother’s womb [1,2]. The term spontaneous miscarriage is used interchangeably with spontaneous abortion or early pregnancy loss [2,3]. In Poland, from 2000 to 2022, there was an upward trend in the occurrence of spontaneous miscarriages, while the total number of pregnancies steadily declined. Currently, the average annual number of lost pregnancies in Poland is 50,187, which constitutes 8.5% of all diagnosed pregnancies. In contrast, this issue affects 12–13.5% of confirmed pregnancies in Scandinavian countries and from 12.5% to 18.7% in the United Kingdom [4,5]. According to the related literature, 80% of cases of spontaneous miscarriage occur during the first trimester [2]. So far, little is known about the aetiology of miscarriages in the second and third trimesters of pregnancy. According to the latest research, there is a correlation between the activity of pro-inflammatory cytokines and late pregnancy loss. The factors that trigger and co-activate signalling pathways leading to the development of inflammation are primarily infections and placental inflammation [6,7]. Additionally, the foetal karyotype may influence the development of inflammation and, therefore, may be attributed to the mechanism of miscarriage [8,9]. Chromosomal abnormalities account for approximately 50–60% of all miscarriages, with a significant portion occurring in the early weeks of pregnancy [10,11].

Causes of miscarriages with unexplained aetiology, despite standard comprehensive diagnostics, should be considered in the context of immunological defects. Full prevention of subsequent reproductive failures should include testing to exclude immunological disorders that contribute to the pathogenesis of early pregnancy loss, which is particularly relevant in cases of recurrent miscarriages. Even highly specialised tests assessing antibody levels and lymphocyte profiles do not provide answers explaining the cause of all miscarriages. Therefore, there is a need to search for other aetiological factors of spontaneous miscarriages related to immune response disorders [3,12]. The results from our previously conducted studies have revealed two somewhat distinct mechanisms of miscarriage related to the formation of NETs (Neutrophil Extracellular Traps) via NETosis and to the disruption of the oxidative–antioxidative balance [13,14]. We have also found that exposure to BPA (Bisphenol A) may affect the course of innate immune mechanisms involving neutrophils [15]. The results of our analyses have not been sufficient to answer the question regarding the actual role of the immunological mechanisms involved in spontaneous miscarriage. Therefore, continuing our research, we have hypothesised the active involvement of the NLRP3 inflammasome (NLR Family Pyrin Domain Containing 3) in the pathogenesis of spontaneous miscarriages.

Inflammasomes are intracellular protein complexes responsible for initiating inflammatory processes in response to bacterial, viral, and fungal infections, as well as cellular stress and tissue damage [16,17,18,19,20]. The activated inflammasome cleaves several proteins, most notably IL-1β and IL-18, but also GSDMD (Gasdermin D), through the activation of caspase-1 [16,17,21]. The activity of caspase-1, which leads to the formation of pores in the cell membrane, results in the loss of the membrane ion gradient and an increase in osmotic pressure due to the influx of water. It leads to cellular swelling and osmotic lysis, ultimately resulting in cell death through pyroptosis, which morphologically distinguishes that process from apoptosis [16,22]. During pyroptosis, caspase-1 cleaves and deactivates metabolic enzymes, while the activation of inflammasomes contributes to the release of inflammatory cytokines [16,17,18,22,23,24].

In recent years, there has been a significant expansion in our understanding of inflammasome activation mechanisms, their structure, and their role in various pathological conditions. It provides hope for utilising this knowledge to develop new therapeutic strategies targeting inflammasomes in medicine, including in prenatal care [17,18,25,26,27,28,29]. It has been demonstrated that the NLRP3 inflammasome signalling pathway affects endometrial receptivity, which is the readiness of the uterine lining to accept and implant the embryo, as well as embryo invasion through the induction of epithelial-to-mesenchymal transition. Therefore, abnormal activation of inflammasomes in the endometrium may adversely affect endometrial receptivity. Excessive activation of the NLRP3 inflammasome mediates an abnormal maternal–foetal inflammatory response and may be associated with pregnancy complications such as preeclampsia, preterm birth, and spontaneous miscarriage [28,29,30,31,32,33,34].

The aim of the conducted research was to evaluate the involvement of the NLRP3 inflammasome as a potential causative factor in the pathogenesis of spontaneous miscarriages. Since the activation of the pro-inflammatory cytokines IL-1β and IL-18 involves the NLRP3 protein platform and caspase-1, the concentrations and expression of those proteins were examined. Studies by other authors have demonstrated a link between inflammasome activation and apoptosis, which is why the quantity and expression of classical apoptosis biomarkers, such as cytochrome C, Fas/FasL proteins, Bcl-2, and p53 protein (which induces apoptosis in response to DNA damage), have been assessed. Given the connection between inflammasomes and NETosis, the obtained results are related to the previous research. The activation of the NLRP3 inflammasome in women with spontaneous miscarriage and pregnant women is associated with disrupted influx or efflux of Ca and K [35,36,37]; therefore, their concentrations, as well as those of Mg and Na, which are important for the proper development of pregnancy, have also been measured [38,39].

## 2. Results

The results of our previous project have led us to analyse potential causes of miscarriage in two groups of women with diagnosed spontaneous miscarriage [13]. The first group included women who had experienced a miscarriage and had not shown NET structures in the placental tissue, and were classified as “NET negative”. The second group comprised women who had experienced a miscarriage and had had NETs present in the placental tissue, and were classified as “NET positive” [13]. Since the currently presented results constitute the continuation of previous studies and hypotheses, we decided to evaluate the obtained data in the group of all women with miscarriage as well as in the NET-negative and NET-positive patient groups.

### 2.1. Measurement of Selected Protein Concentrations by Means of the ELISA Method

Statistically significant higher concentrations of NLRP3 and IL-18 were found in the sera of all women with miscarriage as compared to the values obtained in the control group. Significantly higher median concentrations of IL-18 and IL-1β were observed in the sera of women with NET-negative miscarriages as compared to the control values. Additionally, a significantly higher concentration of IL-1β was found in the sera of women with NET-negative miscarriages as compared to women in the NET-positive group (Table 1).

### 2.2. Measurement of Ca, K, Mg, and Na Concentrations by Means of Spectroscopy

The analysis of the elemental concentrations in whole blood revealed statistically significant lower levels of Ca and K in women with spontaneous miscarriage as compared to the levels found in women with a normally progressing pregnancy. Lower mean concentrations of Ca and K were also observed in the blood of women with NET-negative miscarriages as compared to the values obtained in the control group. Additionally, in the group of women with NET-positive miscarriages, a reduced concentration of Ca in whole blood was found as compared to pregnant women (Table 1).

### 2.3. Assessment of Selected Proteins by Means of Immunohistochemical Methods

The assessment of placental tissue samples revealed a statistically significant higher expression of IL-1β (*p* < 0.0001), IL-18 (*p* < 0.0001), and Caspase-1 (*p* < 0.0001) proteins in all women with miscarriage as compared to the control group. At the same time, a significantly lower expression of FasL (*p* < 0.0001) and Bcl-2 (*p* = 0.008) proteins as well as minimal presence of Ca deposits (*p* < 0.0001) was observed in the placental tissue obtained from women with miscarriage as compared to the tissue from women with a normally progressing pregnancy.

No statistically significant differences were found for NLRP3 and Fas. Expression of p53 protein was not detected in any of the analysed samples from women with spontaneous miscarriage or from the control group.

The detailed analysis of individual cases revealed that 85% of women with miscarriage showed a strong expression of IL-1β, while 15% showed a weak expression, with 100% showing a weak expression in the control group. Expression of IL-18 was observed in 90% of women with miscarriage (45% strong and 45% weak), with no expression in the control group. Placental tissues from women with miscarriage exhibited a high expression of Caspase-1 in 100% of cases and a weak expression in 100% of control women. No expression of FasL protein was detected in the tissue from women with miscarriage, whereas a weak expression of that protein was exhibited by 70% of control samples. A strong expression of Bcl-2 was observed in 31% of women with miscarriage as compared to 40% strong and 40% weak expression in the control group. Only 10% of women with spontaneous miscarriage showed a weak presence of Ca deposits, whereas the control tissue had strong Ca deposits in 60% and weak deposits in 20% of samples. Only 10% of the placental tissue samples from women with miscarriage showed a weak expression of NLRP3 and Fas, with no detection of these proteins in the control samples.

In the NET-negative patients, a significantly higher expression of IL-1β (*p* = 0.0001; 90% with a strong expression), IL-18 (*p* = 0.001; 40% with a strong expression and 40% with a weak expression), and Caspase-1 (*p* = 0.00002; 100% with a strong expression) was observed as compared to the control group. Concurrently, a significantly reduced expression of FasL protein (*p* = 0.002; 100% with no expression) and Ca deposits (*p* = 0.01; 90% with no deposits and 10% with a weak presence of deposits) was noted in comparison to the control samples.

In the NET-positive patient group, similar trends in the expression of the studied parameters were observed. A statistically significant higher expression of IL-1β (*p* = 0.0006; 78% with a strong expression), IL-18 (*p* = 0.0002; 56% with a strong expression and 44% with a weak expression), and Caspase-1 (*p* = 0.00002; 100% with a strong expression) was found as compared to the control group. A significantly lower expression of FasL protein (*p* = 0.002; 100% with no expression) and the presence of Ca deposits (*p* = 0.01; 89% with no deposits and 11% with a weak presence of deposits) were noted in comparison to the controls.

No statistically significant differences were found for the examined parameters in the tissue between the NET-negative and NET-positive patient groups.

**Figure 1:**
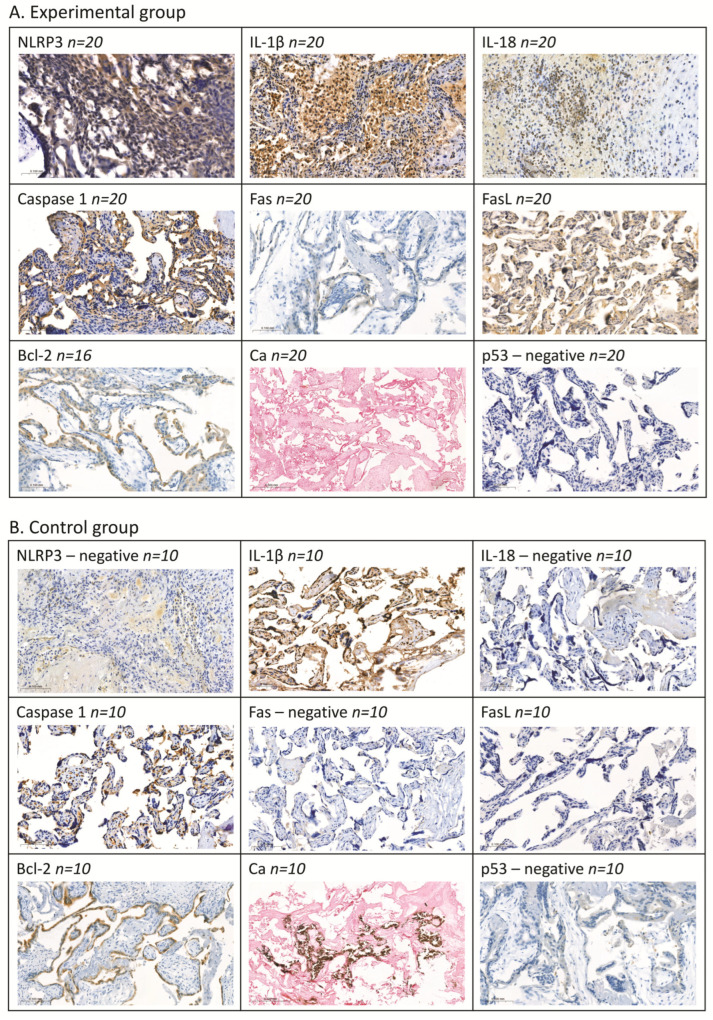
The expression pattern of the studied parameters.

Figure 1 displays a typical expression pattern of the studied parameters.

### 2.4. Correlations

The statistical analysis of the obtained results revealed a relationship between the studied parameters. The correlations are displayed in Table 2.

### 2.5. ROC Curves

In Table 3 and Table 4, the results regarding the diagnostic performance as well as the sensitivity and specificity of the tests for the studied parameters are summarised. The analysis of the results of the blood parameters obtained from the group of all women with spontaneous miscarriage versus the control group demonstrated that the highest diagnostic sensitivity was observed for the Ca parameter (89.04%), while the diagnostic specificity was the highest for NLRP3 and IL-18 (70%). The positive predictive value was the highest for Ca (PPV = 92.86%), and the negative predictive value was the highest for IL-18 (NPV = 38.89%). IL-18 showed the best test performance (AUC = 0.712). Among the studied parameters, only IL-18 (*p* = 0.045) and Ca (*p* = 0.033) demonstrated the a diagnostic performance relative to the cutoff value of AUC = 0.5. IL-18 was observed to have the highest test performance value (AUC = 0.712), diagnostic specificity (70%), and negative predictive value (NPV = 38.89%), while Ca had a higher diagnostic sensitivity (89.04%) and positive predictive value (PPV = 92.86%).

In the group of the NET-negative patients as compared to the control group, IL-18 showed the highest diagnostic sensitivity (72.22%). The diagnostic specificity was the highest for K (80%), the positive predictive value was the highest for Ca (PPV = 88.64%), and the negative predictive value was the highest for IL-18 (NPV = 58.33%). IL-1β achieved the best test performance (AUC = 0.772). A significant diagnostic performance relative to the cutoff value of AUC = 0.5 was demonstrated by IL-1β (*p* = 0.014), IL-18 (*p* = 0.021), Ca (*p* = 0.047), and K (*p* = 0.044). Additionally, NLRP3 also showed a significant diagnostic performance relative to the cutoff value of AUC = 0.5 (*p* = 0.037).

The analysis of the results for the NET-positive group as compared to the control group showed a significant diagnostic performance relative to the cutoff value of AUC = 0.5 exclusively for Ca (*p* = 0.043), with a sensitivity of 57.14%, a specificity of 80%, a positive predictive value (PPV) of 88.89%, and a negative predictive value (NPV) of 40%.

The comparison of the results between the NET-negative group and the NET-positive group revealed that only IL-1β demonstrated a significant diagnostic performance relative to the cutoff value of AUC = 0.5 (*p* = 0.009), with a sensitivity of 92.86%, a specificity of 60%, a positive predictive value (PPV) of 61.90%, and a negative predictive value (NPV) of 92.31%.

## 3. Discussion

The embryo and the woman’s body remain in continuous molecular interaction in a complex process that, besides the trophoblast and decidual tissue, involves several types of cells, primarily of immunological origin. Immunological disorders are becoming an increasingly common cause of difficulties in achieving pregnancy, affecting approximately 10–15% of infertile couples, according to estimates [1,2,3,4,5,12]. A precise understanding of the underlying pathomechanisms of those disorders will allow for their regulation and increase the chances of a successful pregnancy.

The observed increase in serum levels of NLRP3 and IL-18 in the patients with spontaneous abortion suggests a potential involvement of the inflammasome in this obstetric failure. This hypothesis is supported by the high expression of IL-1β and IL-18, along with elevated levels of Caspase-1 in the placenta tissue of women who experienced miscarriage. However, it is noteworthy that elevated levels of NLRP3 expression were found in only 10% of the patients diagnosed with spontaneous abortion. Lu et al. [25] and Gao et al. [26] also observed an increased expression of pro-inflammatory cytokines and the NLRP3 inflammasome in a group of women with recurrent spontaneous abortions. In turn, D’Ippolito et al. [29] and Banerjee et al. [33] demonstrated an increased expression of NLRP3 and pro-inflammatory cytokines in the endometrial tissues of women with idiopathic recurrent spontaneous abortions. The highest diagnostic specificity observed in our studies for NLRP3 and IL-18, with the highest level of test power being found for IL-18, demonstrating the significant diagnostic strength of this cytokine relative to the threshold value, suggests their potential role as biomarkers for miscarriage.

The involvement of increased apoptosis in miscarriage among the patients in our study may be excluded by the low expression of FasL protein and the lack of changes in the Fas receptor expression in the placental tissue. FasL is a transmembrane protein that induces apoptosis through binding to the Fas receptor [40]. Trophoblast cells and connective tissue, which express the FasL protein, induce apoptosis in the activated T cells carrying Fas in the maternal–foetal system. That process helps to minimise the risk of embryo rejection during implantation and early embryo development [40].

The low level of the anti-apoptotic protein Bcl-2 observed in our study in the placental tissue may suggest the local activation of apoptosis. However, the absence of p53 protein expression in the tissue samples from both the study and control groups may indicate a lack of DNA damage that could lead to the activation of apoptosis. Similar results were obtained in the study conducted by Atia T. [41], who measured p53 and Bcl-2 proteins in the placental tissue from women with recurrent miscarriage and spontaneous abortion.

On the other hand, the concurrent lack of changes in cytochrome C levels in the serum of patients with miscarriage may rule out apoptosis. Cytochrome C is released during caspase-independent apoptosis, where calpain, activated by calcium ions, plays an autonomous role. Upon stimulation (e.g., oxidative stress or infection), calcium is released from the endoplasmic reticulum and binds, among other things, to calpain. That enzyme activates pro-apoptotic proteins and releases cytochrome C from the mitochondrion, inducing cell death [42]. The limitation of our preliminary research conclusions, excluding the participation of apoptosis in miscarriage in our patients, is the use of indirect markers of this process. The results require confirmation using TUNEL or cleaved caspase 3 assessment methods.

The probable lack of apoptosis in miscarriage is also indicated by the low levels of calcium ions both in the blood and in the presence of calcium deposits in the placental tissue of women with spontaneous miscarriage. Lower levels of calcium ions in the blood and virtually absent calcium deposits in the placental tissue may result from the excessive outflow of calcium ions from the cell and their direct involvement in a local inflammatory process leading to miscarriage.

The primary function of the placenta is the exchange of gases, energy products, building materials, and their metabolites between maternal blood and foetal blood. Oxygen, carbon dioxide, and most minerals diffuse across the placenta. Additionally, glucose, amino acids, and calcium and potassium ions are actively transported through the placenta. In the third trimester of pregnancy, calcium ions are transferred intensively across the placenta due to the ongoing mineralisation of the foetal skeleton. Over time, the placenta undergoes ageing processes, including the occlusion of placental blood vessels, calcium deposition in the villi, fatty infiltration, and fibrosis. This may pose a risk to the foetus, particularly in post-term pregnancies [43,44,45].

It has been demonstrated that the activation of the NLRP3 inflammasome and Caspase-1, along with the release of inflammatory cytokines (IL-1β and IL-18), occurs as a result of intense calcium and potassium efflux through the membrane integrity disruption in the cell, leading to mitochondrial damage and the generation of reactive oxygen species (ROS) [18,28,37,46,47]. A common factor observed in all our patients with spontaneous miscarriage and in the NET-negative group was a significantly reduced level of Ca and K in whole blood. The highest diagnostic sensitivity for Ca, along with its positive predictive value and significant diagnostic power relative to AUC, demonstrated in our statistical analysis indicates that this element may be useful in the early diagnosis of miscarriage risk. This is supported by the moderately strong association between the NLRP3 inflammasome concentration and Ca in women with spontaneous miscarriage and in the “NET-negative” group. Additionally, in the group of women with diagnosed spontaneous miscarriage, we found a similar statistical relationship between NLRP3 and Na. An interesting finding is the strong negative correlation between IL-18 levels and Ca in the “NET-negative” group, suggesting an intense loss of Ca ions and the release of IL-18 immediately following the activation of the NLRP3 inflammasome.

The difficulty in interpreting the results for Na and Mg, which were found at lower levels in the whole blood of women with spontaneous miscarriage, arises from the high individual variability and the absence of reference ranges. Additionally, diet and place of residence significantly influence the levels of these elements in pregnant women at all stages of pregnancy [4,14]. Pathological states in the human body have a minimal impact on the concentration of elements in plasma/serum but significantly disrupt the homeostasis of intracellular ionised elements. The measurement of ionised elements within cells has a significantly higher diagnostic value as compared to plasma measurements [48]. Comparing the levels of selected elements, we observed much lower concentrations of potassium (K) and sodium (Na) in the blood of both groups of women we studied, as compared to the results obtained by Kot et al. [38] in women with normally progressing pregnancies. At the same time, we recorded higher concentrations of calcium (Ca) and magnesium (Mg) in both groups of our patients as compared to those in studies by Kot et al. [38] and Gong et al. [49]. However, it should be noted that the literature and prenatal care standards lack recommended reference ranges for ionised minerals measured in the whole blood of pregnant women when taking into account pregnancy trimesters and different pathological conditions of pregnancy. When compared to the reference ranges established for adults [50], the concentrations of the minerals we analysed (Ca, K, and Na) in the blood of women with spontaneous abortion were found to be below the lower limit. Only in the case of Mg did we observe an increase in concentration in all the groups of women we studied as compared to the ionogram accepted for the adult population. Magnesium and calcium are natural antagonists; this relationship was clearly observed in all the groups of women in our study [50].

Water–electrolyte disturbances, which are very common during pregnancy, particularly in its early stages, manifest as symptoms such as nausea and vomiting, general weakness, headaches, irritability, painful muscle cramps and weakness, heart rhythm disturbances, and elevated blood pressure. These symptoms may both result from and contribute to spontaneous abortion or other pregnancy pathologies [35,38,39,51]. The observed strong or moderately strong positive correlations between levels of Ca and levels of K and Na in the three analysed groups of women with spontaneous abortion suggest disturbances in the electrolyte balance that could be either a cause or a consequence of foetal loss.

The statistical analysis of our results, considering the two patient groups, revealed that significant changes in IL-1β and IL-18 levels, as well as higher NLRP3 levels, are characteristic of the NET-negative patients. This finding excludes the involvement of the inflammasome in the formation of NETs. On the other hand, the high expression of inflammasome activation biomarkers in the placental tissue observed in both the NET-negative and NET-positive women suggests a common factor for those two groups of patients. However, the higher IL-1β levels in the sera of NET-negative patients as compared to the NET-positive patients suggest intensified inflammatory processes independent of NETs and may be useful for differentiating between these patients. The results obtained from the tissues of the NET-positive patients indicate the local activation of the inflammasome, potentially involving other cells. The lack of differences between the NET-negative and NET-positive groups may be due to small sample sizes. It is also possible that NETosis and autophagy occur independently in the placental tissue.

The activation of the NLRP3 inflammasome, caused among other factors by membrane damage, results in the formation of reactive oxygen species (ROS) under conditions of cellular stress, due to mitochondrial damage [17,21,52,53,54,55,56,57]. In the context of the previously obtained results [14], the current data suggest the involvement of the inflammasome in the processes related to the disruption of the oxidative–antioxidant balance during pregnancy loss. Oxidative stress is an inherent process occurring in a pregnant woman’s body. Disruption of the pro-/antioxidant balance may lead to pregnancy loss [14,58]. Li et al. [27] observed an increased expression of the NLRP1 protein and excessive autophagy under oxidative stress in the HTR-8/SVneo cell line (trophoblast cells). They demonstrated that NLRP1 inflammasome activation and autophagy were interrelated and influenced one another. The NLRP1 activator, belonging to the inflammasome complex family, may significantly increase the expression of both NLRP1 and NLRP3 proteins [27]. The NLRP1 inflammasome, similar to NLRP3, plays a crucial role in the pathogenesis of inflammation. The authors also demonstrated the increased expression of the following proteins: pro-CASP1, CASP1, pro-IL-1β, and IL-1β [27]. According to Li et al. [27] and Mulla et al. [34], healthy trophoblast cells early in pregnancy may activate the NLRP3 inflammasome and increase the expression of IL-1β, leading to an abnormal inflammatory response associated with pregnancy loss.

**Figure 2:**
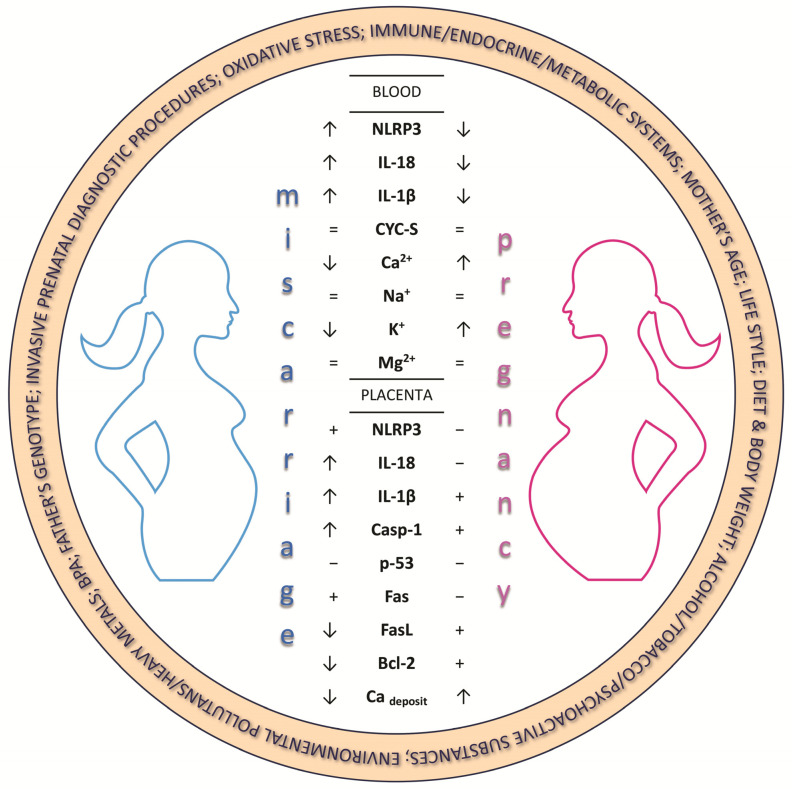
Proposed cause–effect model in women with miscarriage and pregnant women, based on the obtained research results. The course of pregnancy is significantly influenced by many external and internal factors, the negative effect of which may be miscarriage. The most important of them include environmental pollution/heavy metals; exposure to BPA; invasive prenatal diagnostic procedures; paternal genotype; oxidative stress; activity of the immune/endocrine/metabolic system of the mother; maternal age and lifestyle; diet and body weight; and consumption of alcohol/tobacco/psychoactive substances. In addition, the significantly higher levels of NLRP3, IL-18, and IL-1β and lower amounts of Ca^2+^ and K^+^ in women with miscarriage compared to women with a normal pregnancy suggest the participation of these molecules in the processes leading to miscarriage. NLRP3—NLR Family Pyrin Domain Containing 3; IL-18—Interleukin 18; IL-1β—Interleukin 1 Beta; CYC-S—Cytochrome C Somatic; Ca^2+^—calcium ions; Na^+^—sodium ions; K^+^—potassium ions; Mg^2+^—magnesium ions; Casp-1—Caspase 1; p53—tumor protein p53; Fas—Fas receptor; FasL—Fas Ligand Protein; Bcl-2—B-cell lymphoma 2 protein; Ca deposit—calcium deposits; **↑**—increase in concentration/expression; **↓**—decrease in concentration/expression; =—similar quantities; +—expression; −—no expression.

The lack of a strong correlation and the presence of only moderately strong associations between the parameters we studied are likely due to the small sample sizes. Nevertheless, the existence of relationships between the analysed parameters indicates that the proposed research panel is appropriate and could be recommended in the future for pregnant women and those planning to conceive as part of preventive prenatal care (Figure 2).

The limited number of similarly themed studies by other authors complicates the analysis of our results. However, our data already suggest that prophylaxis, prompt diagnosis, and targeted treatment of the NLRP3 inflammasome may soon represent a breakthrough in preventing spontaneous miscarriages, much like what is happening with the treatment of many other diseases [18,19,22].

Establishing a new testing panel that includes the aforementioned parameters as part of prenatal care should be a priority, especially in light of the continually decreasing number of pregnancies and the increasing rate of reproductive failures.

## 4. Materials and Methods

### 4.1. Study and Control Group

The study group consisted of 84 patients who had experienced spontaneous miscarriage between the 4th and 19th week of pregnancy, aged 18–44 years. The patients were hospitalised at the Department of Obstetrics and Perinatology of the University Clinical Hospital in Białystok or the Obstetrics Department with Pregnancy Pathology of the Jędrzej Śniadecki Voivodeship Hospital Complex in Białystok. The women included in the study group did not have any other underlying diseases; antiphospholipid syndrome and venous thrombosis were also excluded. The control group consisted of 14 healthy women, aged 18–32 years, who had had normal pregnancies and given birth to healthy children. The women in the control group were carefully selected, excluding all chronic and temporary diseases throughout the pregnancy. The control group included women who already had children (0–3 children).

### 4.2. Materials

The study input matter consisted of blood samples collected from the cubital vein and fragments of the placental tissue, obtained immediately after miscarriage from women in the study group (4th to 19th week of pregnancy). In the control group, blood was collected during routine tests in the first trimester of a normal pregnancy (6th to 14th week). The placental tissue from the control group women was obtained after childbirth. Each woman participating in the study provided written consent for sampling.

Sampling took place during the project “The Impact of Diet and Smoking on the Levels of Zinc, Selenium, Copper, Manganese, and Antioxidant Status in Women with Spontaneous Miscarriage” funded by a promoter’s grant from the Ministry of Science and Higher Education (grant number: N N405 625538, Ethics Committee approval number R-I-002/348/2007). The samples were stored at −80 °C in accordance with the Good Laboratory Practice principles. The approval for the expansion of the analysis panel in respect of specific samples was obtained from the Bioethics Committee of the Medical University of Białystok under the numbers APK.002.423.2020 and APK.002.429.2022. Due to the varied amounts of material in the available samples, not all the tests were performed on every sample in the entire group.

### 4.3. Methods

#### 4.3.1. Determination of Selected Proteins by Means of the Enzyme-Linked Immunosorbent Assay (ELISA) Method

The concentrations of NLRP3 (NLR Family Pyrin Domain Containing 3), IL-1β (Interleukin 1 Beta), IL-18 (Interleukin 18), and CYCS (Cytochrome C) proteins were measured in blood serum using commercial Enzyme-Linked Immunosorbent Assay Kits from Cloud-Clone Corp. (Katy, TX, USA) according to the manufacturer’s instructions.

**Figure 3:**
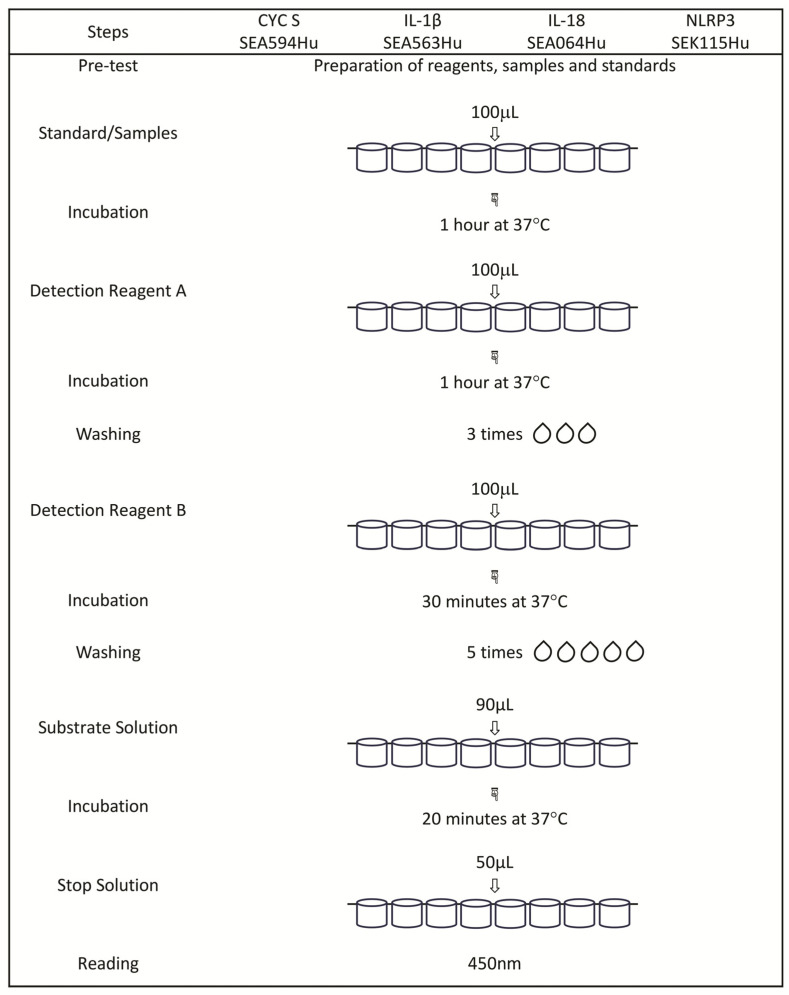
The abbreviated protocols for the procedures of the performed assays.

The abbreviated protocols for the procedures of the performed assays are presented in Figure 3.

#### 4.3.2. Determination of Ca, K, Mg, and Na by Means of the ICP-OES Method

**Figure 4:**
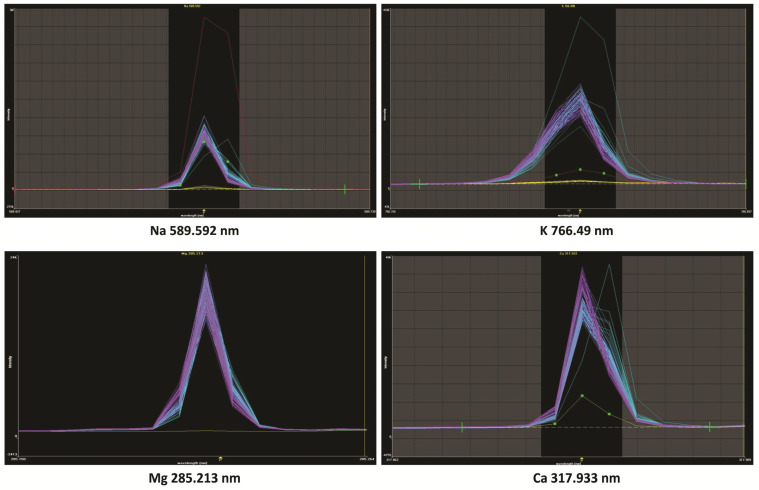
The emission signal profiles of the analysed elements in the samples.

The analysis of whole-blood samples for Ca, K, Mg, and Na content was carried out by means of the Optima 8000 (PerkinElmer, Waltham, MA, USA) ICP-OES spectrometer (Inductively Coupled Plasma–Optical Emission Spectroscopy) with dual observation of plasma (axial and radial). The spectrometer was equipped with a cyclonic spray chamber with concentric Mira-mist nebuliser. Specifications and working conditions of the ICP-OES spectrometer are presented in Table 5. All of the samples were placed on Ratek roller mixer (Lab Dencer, IKA, Staufen i. Breisgau, Germany) to ensure complete homogenisation of samples. An aliquot of 0.25 mL of whole blood was added to a 25 mL quartz microwave digestion vessel with a Teflon screw cup. A total of 3 mL of concentrated nitric acid (Suprapur, Fluka, Neu-Ulm, Germany) was added and given 30 min of pre-reaction time in the fume hood. After that, the sample was sealed, placed in the microwave digestion system (SpeedWave Two, Berghoff, Münster, Germany), and digested following the programme described in Table 6. At the end of digestion, the sample was removed from the microwave and allowed to cool to room temperature. Next, the sample was quantitatively transferred to the acid-washed polypropylene tube and diluted to the final volume with deionised water. All of the samples were stored in a freezer at the temperature of −20 °C until the analysis commencement. The emission signal profiles of the analysed elements in the samples are presented in Figure 4.

#### 4.3.3. Analysis of Selected Proteins by Means of Immunohistochemical Methods

In the placental tissues, the proteins NLRP3, IL-1β, IL-18, Caspase-1, Fas, FasL, Bcl-2, and p53 were measured by means of immunohistochemical techniques. Tissues containing paraffin blocks were cut using a microtome into 4 μm thick sections and placed on silanised slides. The sections were deparaffinised with xylene and hydrated through a series of alcohol washes. Next, the sections were placed in citrate buffer (pH = 6.0) and incubated in an aqueous bath for 20 min at 98.5 °C to reveal the antigen and were subsequently incubated for 20 min at room temperature. The sections were subsequently incubated with 3% hydrogen peroxide to block endogenous peroxidase and with 1% bovine serum to block nonspecific bonds. In the next stage, the slides were incubated with specific antibodies: anti-NLRP3 (dilution 1:100, Sigma Aldrich, SAB5700723, St. Louis, MO, USA), anti-IL-1β (dilution 1:300, orb420045, Biorbyt, Wuhan, China), anti-IL-18 (dilution 1:200, HPA003980, Sigma Aldrich, USA), anti-CASP1 (dilution 1:300, HPA003056, Sigma Aldrich, USA), Fas (dilution 1:100, HPA027444, Sigma Aldrich, USA), FasL (dilution 1:100, HPA054959, Sigma Aldrich, USA), Bcl-2 (dilution 1:100, sc-7382, C-2, Santa Cruz Biotechnology, Dallas, TX, USA), and p53 (dilution 1:100, Pab 1801, sc-98, Santa Cruz Biotechnology, USA) for 30 min at room temperature. After a reaction was induced through the use of a polymeric technique (Immunohistochemistry Application Solutions Kit, #13079, Cell Signalling, Danvers, MA, USA), the antigen–antibody complex was exposed by incubation to chromogen 3.3′-diaminobenzidine (SignalStain^®^ DAB Substrate Kit, Cell Signaling, USA). The cellular nuclei were stained with haematoxylin. The positive staining percentage for proteins was determined by counting the number of cells staining positively in the syncytiotrophoblast and classified into 3 groups by semiquantitative evaluation: 2—positive expression ≥11% of cells; 1—weak expression max. 10% of cells; and 0—no expression. The evaluation of immunohistochemical staining was performed with a light microscope using 200× and 400× magnification (Olympus BX41 Clinical Microscope, Pottstown, PA, USA).

#### 4.3.4. Determination of Calcium Deposits Using the Von Kossa Method

A Von Kossi staining kit (Abcam, ab150687, Cambridge, UK) was used for visualising calcium deposits in the placenta tissue. Dusty calcifications were observed and classified as 0—no calcifications, both scattered and in deposits; 1—one single calcification in the intervillous subsite of placental parenchyma (PP); and 2—multiple calcifications in the intervillous subsite of placental parenchyma (PP).

#### 4.3.5. Statistical Analysis

The statistical analysis was conducted by means of PQStat Software v.1.8.4, Poznań, Poland. The normality of the distribution of the studied parameters was assessed by means of the Shapiro–Wilk test. The parameters NLRP3, Ca, K, Mg, and Na were evaluated by means of the parametric Student’s t-test for the purpose of comparisons between two groups, and ANOVA with Tukey’s post hoc test for the purpose of comparisons among more than two groups. The parameters CYCS, IL-1β, and IL-18 were analysed by means of the non-parametric Mann–Whitney U test for the purpose of comparisons between two groups, and the Kruskal–Wallis ANOVA with Conover–Iman post hoc test for the purpose of comparisons among more than two groups. Tissue parameters (anti-NLRP3, anti-IL-1β, anti-IL-18, anti-CASP1, Fas, FasL, Bcl-2, p53, and Ca deposits) were assessed by means of Fisher’s exact test and Fisher’s exact test with Benjamini–Hochberg correction for multiple comparisons. Correlation assessment was conducted using Pearson’s r test for normally distributed parameters and Spearman’s r test for parameters without a normal distribution.

The assessment of diagnostic reliability (sensitivity and specificity, positive and negative predictive values, and diagnostic power of the test) was performed by means of the ROC curve and the area under the ROC curve (AUC). Optimal cutoff points were determined by applying the method of minimal distance from the upper-left corner and compiled to be presented in Table 7.

## Figures and Tables

**Table 1:** Summary of the blood results.

*Non-parametric tests*				*Parametric tests*						
**Experimental group**										
Parameter	Serum			Parameter	Serum	Parameter	Whole blood			
	**CYC S** [ng/mL] *n* = 32	**IL-1β** [pg/mL] *n* = 33	**IL-18** [pg/mL] *n* = 31		**NLRP3** [ng/mL] *n* = 44		**Na** [mmol/L] *n* = 66	**K** [mmol/L] *n* = 59	**Mg** [mmol/L] *n* = 65	**Ca** [mmol/L] *n* = 73
Median	1.485	15.23	**19.15** *	Mean	**0.133** *	Mean	3.622	**3.125** *	1.569	**2.120** *
Q1	1.382	13.39	14.05	SD	0.068	SD	0.220	0.280	0.130	0.187
Q3	1.562	46.00	37.19	-	-	-	-	-	-	-
Min.	1.331	9.122	7.929	Min.	0.038	Min.	3.027	2.175	1.280	1.754
Max.	1.946	95.24	75.62	Max.	0.295	Max.	4.035	3.485	1.761	2.483
**“NET-negative” group**										
Parameter	Serum			Parameter	Serum	Parameter	Whole blood			
	**CYC S** [ng/mL] *n* = 23	**IL-1β** [pg/mL] *n* = 22	**IL-18** [pg/mL] *n* = 18		**NLRP3** [ng/mL] *n* = 30		**Na** [mmol/L] *n* = 41	**K** [mmol/L] *n* = 37	**Mg** [mmol/L] *n* = 41	**Ca** [mmol/L] *n* = 45
Median	1.510	**34.01 ^ac^**	**23.93 ^a^**	Mean	0.137	Mean	3.633	**3.107 ^a^**	1.563	**2.114 ^a^**
Q1	1.421	14.09	15.46	SD	0.064	SD	0.226	0.268	0.125	0.193
Q3	1.562	50.26	57.04	-	-	-	-	-	-	-
Min.	1.331	10.28	9.554	Min.	0.043	Min.	3.027	2.367	1.280	1.808
Max.	1.690	91.40	75.62	Max.	0.163	Max.	4.019	3.485	1.761	2.483
**“NET-positive” group**										
Parameter	Serum			Parameter	Serum	Parameter	Whole blood			
	**CYC S** [ng/mL] *n* = 9	**IL-1β** [pg/mL] *n* = 11	**IL-18** [pg/mL] *n* = 13		**NLRP3** [ng/mL] *n* = 14		**Na** [mmol/L] *n* = 25	**K** [mmol/L] *n* = 22	**Mg** [mmol/L] *n* = 24	**Ca** [mmol/L] *n* = 28
Median	1.408	13.58	16.86	Mean	0.123	Mean	3.605	3.155	1.581	**2.130 ^b^**
Q1	1.357	11.06	10.86	SD	0.076	SD	0.213	0.304	0.140	0.180
Q3	1.536	15.13	32.29	-	-	-	-	-	-	-
Min.	1.357	9.122	7.929	Min.	0.038	Min.	3.245	2.175	1.291	1.754
Max.	1.946	95.24	43.57	Max.	0.295	Max.	4.035	3.477	1.760	2.480
**Control group**										
Parameter	Serum			Parameter	Serum	Parameter	Whole blood			
	**CYC S** [ng/mL] *n* = 5	**IL-1β** [pg/mL] *n* = 10	**IL-18** [pg/mL] *n* = 10		**NLRP3** [ng/mL] *n* = 5		**Na** [mmol/L] *n* = 9	**K** [mmol/L] *n* = 11	**Mg** [mmol/L] *n* = 11	**Ca** [mmol/L] *n* = 10
Median	1.536	13.15	13.40	Mean	0.069	Mean	3.776	3.359	1.644	2.300
Q1	1.421	11.93	9.230	SD	0.067	SD	0.304	0.387	0.139	0.308
Q3	1.728	18.36	22.59	-	-	-	-	-	-	-
Min.	1.305	10.67	6.084	Min.	0.008	Min.	3.440	2.824	1.379	1.937
Max.	1.921	31.10	33.59	Max.	0.137	Max.	4.336	4.083	1.885	2.957
*p*-value										
		^a^ 0.0207 ^c^ 0.0409	* 0.0457 ^a^ 0.0141		* 0.0427			* 0.0196 ^a^ 0.0169		* 0.0108 ^a^ 0.0114 ^b^ 0.0278

Min.—minimum; Max.—maximum; Q1—first quartile; Q3—third quartile; SD—Standard Deviation.; *—statistically significant difference between experimental vs. control; ^a^—statistically significant difference between NET-negative vs. control groups; ^b^—statistically significant difference between NET-positive vs. control groups; ^c^—statistically significant difference between NET negative vs. NET positive; parametric tests: Student’s *t*-test for comparisons of two groups, ANOVA with post hoc Tukey test for comparisons of more than two groups; non-parametric tests: Mann–Whitney U for comparisons of two groups, Kruskal–Wallis ANOVA test with Conover–Iman post hoc test for comparisons of more than two groups.

**Table 2:** Correlations between assessed parameters.

Group	Correlated Parameters	*p*	r
**Experimental**	NLRP3 vs. Ca	*0.0198*	0.3501
	NLRP3 vs. Na	*0.0081*	0.394
	Na vs. Ca	*0.0078*	0.3298
**“NET-negative”**	NLRP3 vs. Ca	*0.022*	0.456
	IL-18 vs. Ca	*0.028*	−0.517
**“NET-positive”**	K vs. Ca	*0.0227*	0.5192
	Na vs. Ca	*0.0301*	0.4342

*p*—*p*-value; r—correlation coefficient.

**Table 3:** Results of the analysis of the diagnostic power of the test.

DeLong’s Method	Serum				Whole Blood			
Parameter	CYC S	IL-1β	IL-18	NLRP3	Na	K	Mg	Ca
*Women with miscarriage* vs. *control*								
AUC	0.541	0.680	0.712	0.646	0.641	0.636	0.639	0.708
SE (AUC)	0.287	0.086	0.095	0.093	0.112	0.100	0.090	0.097
−95% CI	0	0.511	0.525	0.462	0.420	0.438	0.461	0.517
+95% CI	1	0.849	0.900	0.830	0.862	0.833	0.816	0.899
Z	0.235	1.710	2.003	1.436	1.369	1.428	1.468	2.126
*p*	0.813	0.087	*0.045*	0.150	0.170	0.153	0.141	*0.033*
*NET negative* vs. *control*								
AUC	0.543	0.772	0.766	0.728	0.626	0.720	0.674	0.702
SE (AUC)	0.293	0.083	0.095	0.089	0.119	0.098	0.094	0.097
−95% CI	0	0.609	0.579	0.552	0.391	0.526	0.488	0.510
+95% CI	1	0.936	0.953	0.903	0.860	0.913	0.859	0.893
Z	0.240	2.439	2.301	2.081	1.174	2.008	1.758	1.985
*p*	0.809	*0.014*	*0.021*	*0.037*	0.240	*0.044*	0.078	*0.047*
*NET positive* vs. *control*								
AUC	0.537	0.504	0.638	0.696	0.666	0.590	0.579	0.717
SE (AUC)	0.283	0.135	0.121	0.187	0.112	0.116	0.105	0.106
−95% CI	0	0.239	0.400	0.328	0.445	0.362	0.372	0.508
+95% CI	1	0.769	0.876	1	0.888	0.819	0.786	0.927
Z	0.184	0.035	1.116	1.168	1.463	0.840	0.746	2.022
*p*	0.853	0.971	0.264	0.242	0.143	0.400	0.455	*0.043*
*NET negative* vs. *NET positive*								
AUC	0.642	0.766	0.668	0.577	0.538	0.582	0.549	0.523
SE (AUC)	0.129	0.081	0.099	0.101	0.075	0.078	0.079	0.070
−95% CI	0.389	0.605	0.474	0.378	0.390	0.427	0.394	0.385
+95% CI	0.895	0.926	0.863	0.776	0.686	0.736	0.705	0.660
Z	1.236	2.606	1.581	0.818	0.522	1.050	0.666	0.328
*p*	0.216	*0.009*	0.113	0.412	0.601	0.293	0.505	0.742

AUC—Area Under the Curve; SE—Standard Error; CI—Confidence Interval; Z—Z-scores; *p*—*p-*value.

**Table 4:** Diagnostic sensitivity and specificity of the tested parameters.

Parameter	Sensitivity	Specificity	PPV	NPV
*Women with miscarriage* vs. *control*				
**IL-18**	64.52%	**70.00%**	86.96%	**38.89%**
**NLRP3**	54.55%	**70.00%**	88.89%	25.93%
**K**	55.93%	63.64%	89.19%	21.21%
**Ca**	**89.04%**	50.00%	**92.86%**	38.46%
*NET negative* vs. *control*				
**IL-1β**	68.18%	70.00%	83.33%	50.00%
**IL-18**	72.22%	70.00%	81.25%	**58.33%**
**K**	56.00%	**80.00%**	87.50%	42.11%
**Ca**	**86.67%**	50.00%	**88.64%**	45.45%
*NET positive* vs. *control*				
**Ca**	57.14%	80.00%	88.89%	40.00%
*NET negative* vs. *NET positive*				
**IL-1β**	92.86%	60.00%	61.90%	92.31%

PPV—positive predictive value; NPV—negative predictive value.

**Table 5:** Specifications and working conditions of ICP-OES spectrometer.

Optical System	Polichromator Eschelle
Wavelength	Ca 317.333 nm
	Mg 285.213 nm
	K 766.49 nm
	Na 589.592 nm
Detector	Semiconductor device (SCD)
RF generator power	1.3 kW
Radio frequency	27.12 MHz
Plasma observation	Radial
Pomp rate	1 mL/min
Integration time	5 s
Spray chamber	Cyclonic with concentric Mira-mist nebuliser
Gas flow	auxiliary 0.2 L/min
	plasma 13 L/min
	nebulizer 0.55 L/min

**Table 6:** Microwave digestion programme.

Step	Power (W)	Ramp Time (s)	Hold Time (min)
1	800	15	10
2	0	-	15

**Table 7:** Optimal cut-off points.

Material	Serum				Whole Blood				Placental Tissues							
Parameter	CYC S	IL-1β	IL-18	NLRP3	Na	K	Mg	Ca	NLRP3	IL-1β	IL-18	Casp1	Fas	FasL	Bcl-2	Ca Deposit
Optimal cut-off for the diagnostic test	1.510	15.23	16.43	0.126	3.598	3.118	1.615	2.302	1	2	1	2	-	0	0	0
Optimal cut-off for the differential test	1.433	31.95	18.53	0.121	3.671	3.179	1.649	2.193	0	2	2	-	-	-	0	1

## Data Availability

The original contributions presented in the study are included in the article, further inquiries can be directed to the corresponding author.
